# R-CHOP versus R-CVP in the treatment of follicular lymphoma: a meta-analysis and critical appraisal of current literature

**DOI:** 10.1186/1756-8722-2-14

**Published:** 2009-03-24

**Authors:** Ganguly Siddhartha, Patel Vijay

**Affiliations:** 1 Division of Hematology/Oncology, Department of Medicine, University of Kansas Medical Center, Kansas City, Kansas, USA

## Abstract

**Purpose:**

R-CHOP (rituximab with cyclophosphamide, doxorubicin, vincristine and prednisone) and R-CVP (rituximab with cyclophosphamide, vincristine and prednisone) have both been used successfully in the treatment of patients with symptomatic follicular lymphoma (FL). No study has compared the efficacy of the two treatment modalities and attempted to evaluate the role of anthracyclines in the management of patients with FL. We conducted a meta-analysis of relevant literature comparing the two treatment arms for FL with response being the final endpoint.

**Patients and Methods:**

Two analyses were conducted: The first analysis compared R-CHOP to R-CVP as frontline agents for the treatment of FL, and the second analysis included both untreated and relapsed patients.

**Results:**

For both studies, R-CVP was superior to R-CHOP when evaluating for complete response (CR). Odds ratios were 2.86 (95% CI, 1.81–4.51) in the first analysis and 1.48 (95% CI, 0.991–2.22) in the second analysis. However for overall response (CR+Partial response, PR), R-CHOP was superior, with odds ratios of 5.45 (95% CI: 2.51 – 11.83) and 5.54 (95% CI: 2.69 – 11.40), for the first and second analyses, respectively.

**Conclusion:**

R-CHOP and R-CVP protocols achieve excellent overall response. In patients with known cardiac history, omission of anthracyclines is reasonable and R-CVP provides a competitive CR rate. In younger patients with FL where cumulative cardio-toxicity may be of importance in the long term and in whom future stem cell transplantation is an option, again R-CVP may be a more appealing option.

## Introduction

Follicular lymphomas (FL) are for the most part indolent B-cell non-Hodgkin's lymphomas (B-NHL). Median survival is 9 to 11 years. Though FL initially responds to combination and single-agent chemotherapy, the disease ultimately relapses, with no plateau in the survival curve. While cyclophosphamide, doxorubicin, vincristine and prednisone (CHOP) [1] has been the initial chemotherapy of choice for patients with aggressive NHL, no such standard exists for patients with FL. Rituximab, a monoclonal antibody to CD20 antigen, is now commonly added to chemotherapy regimens for FL. Rituximab has been shown to have a favorable toxicity profile and to significantly increase time to progression (TTP) and response rates when used as a single agent in the treatment of symptomatic FL [2]. Given such encouraging results, Czuczman et al. treated FL patients with a combination of rituximab and CHOP (R-CHOP) [3]. Updated results showed that the overall response rate was 100%; with 87% of patients achieving a complete response or unconfirmed complete response [4]. The median TTP and duration of response was 82.3 months and 83.5 months, respectively. Hiddemann et al. reported a large prospective study comparing R-CHOP directly to CHOP in patients with FL [5]. They found that R-CHOP reduced the relative risk of treatment failure by 60% and significantly prolonged time-to-treatment-failure when compared to CHOP. Domingo-Domenech et al. reported an overall response rate of 88% in patients with relapsed FL who were treated with R-CHOP [6]. Marcus et al. compared rituximab, cyclophosphamide, vincristine, prednisone (R-CVP) vs. CVP alone and found an 81% response and 47% complete response for R-CVP vs. 57% and 10% for CVP [7]. Based on the existing literature, R-CHOP or R-CVP has become the standard of care for the treatment of patients with symptomatic advanced FL. Hainsworth et al.[8] used R-CVP or R-CHOP, depending on the patients' cardiac co-morbidities, and showed a 93% response rate with 55% complete remission and prolonged progression-free survival. However the authors did not isolate and compare the results for R-CVP vs. R-CHOP. Moreover, one may be reasonably concerned about the long-term risk of cumulative cardiac toxicities when using doxorubicin (an anthracycline) in patients with indolent lymphoma.

To our knowledge, there has been no head-to-head comparison of the efficacy of R-CVP vs. R-CHOP in patients with FL. We do know that treatment with CHOP is significantly more expensive than with CVP [9]. Considering its greater cost and its potential for causing long-term cardiac toxicities, R-CHOP would therefore seem to be less attractive than R-CVP for treating FL. However, a significant difference in efficacy favoring R-CHOP-if such were shown to exist might outweigh these factors. It is therefore important to assess the relative efficacy of the two treatments.

Our first analysis reviewed the studies of frontline treatment of patients with FL using either R-CVP or R-CHOP. There are no published data illustrating R-CVP as a therapeutic modality for relapsed or previously treated patients with FL, so it is impossible to compare responses to R-CVP and R-CHOP in these patients. With this in mind, in a second analysis we attempted to compare response rates for R-CHOP and R-CVP in patients with FL irrespective of the previous treatment status.

## Patients and Methods

### Data sources

Following the method of Falagas et al. [10], we did a systematic literature search involving Pubmed, the Cochrane Central Register of Controlled Trials databases, and the American Society of Hematology's (ASH) abstract collection, as well as references cited in relevant articles. Search terms included "Follicular lymphoma", "Rituximab", "R-CHOP", and "R-CVP."

### Study selection

The articles were analyzed and relevant literature was further evaluated. Published and unpublished data as well as ASH abstracts were reviewed. No attempt was made to judge studies' scientific merits (for example using the QUOROM algorithm) [11]. A study was considered acceptable if it prospectively evaluated the effectiveness of R-CHOP or R-CVP, alone or in combination with another treatment, for treating follicular lymphoma. To be considered eligible, the data from each treatment arm had to have been reported separately and had to be extractable. No restrictions were imposed on language, journal type, or publication date. A total of seven studies were considered. Four of those met all inclusion criteria as studies of frontline or re-treatment. Two of those four met the criteria for the first analysis (frontline treatment alone).

### Outcome

The primary goal of the analyses was to compare the response rates of R-CVP and R-CHOP in the treatment of patients with FL as frontline therapy or as re-treatment in patients with relapsed disease. In every study patients were classified as having no response, partial response (PR) or complete response (CR). The definitions of tumor response used in two of the studies were in accordance with the International Working Group Criteria [12], while one study used its own criteria for quantifying response. For the present analysis we used three categories: Complete Response: CR; Partial Response: PR, Total Response: CR+PR, the last being the sum of the first two.

Other end points, such as time to treatment failure, duration of response and survival, were looked at, but not directly compared due to inter-study variation in reporting.

### Statistical analysis

Statistical analyses were performed using SPSS 14.0 (SPSS, Chicago Ill). Patients' demographics and clinical characteristics were expressed as proportions and compared across treatments using 2-tailed Chi-squared tests. Treatment efficacy was expressed as proportions of CR, PR and CR+PR, and compared across treatments using 2-tailed Chi-squared tests and odds ratios with 95% confidence intervals (CI). *P *values < 0.05 were considered statistically significant.

## Results

### Selected studies

**Figure 1:**
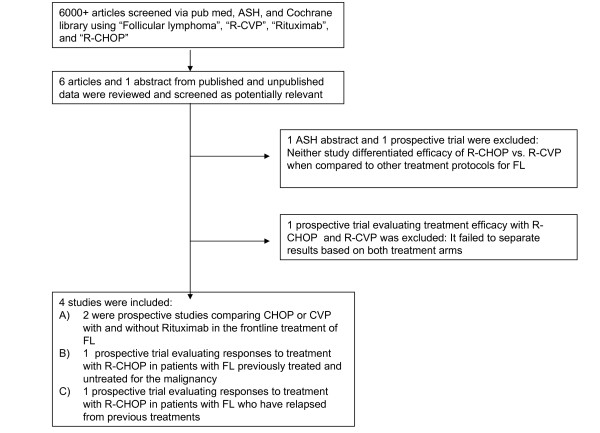
**Article review process**.

In figure 1 is presented the flow diagram showing the steps we took to identify relevant literature for our two analyses. After review of all available literature (all languages), three published studies were considered appropriate for the first analysis [5-7] and four for the second [4-7]. Two studies involved randomized controlled trials comparing treatment protocols of either R-CHOP vs. CHOP or R-CVP vs. CVP. The other two studies were non-randomized clinical trials which were designed to show response, as previously described, by patients with FL treated with R-CHOP. All four of the studies included R-CVP or R-CHOP as the chemotherapeutic regimen to treat FL and had relevant endpoints of CR or PR for all stages of follicular lymphoma.

### Patient characteristics

Demographics are shown in tables 1 and 2. The first analysis, in which R-CVP and R-CHOP are compared as frontline chemotherapeutic agents, included the R-CHOP study of Hiddemann et al. [5] and the R-CVP study of Marcus et al. [7]. Both of these provided adequate patient data; the comparison is shown in table 1. For our second analysis, we compared R-CVP with R-CHOP as a frontline or repeat treatment in patients with FL. Patient characteristics in the second analysis are presented in table 2. Sex, age and stage were the only patient characteristics reported in all four studies. The studies by Hiddemann et al. [5] and Domingo-Domenech et al. [6] included patients with FL of stages 3 and 4 but did not show data from each stage separately. Czuczman et al. [4] and Marcus et al. [7] did not include patients of stage 1. To make the data accurate and comparable across all four studies, we reassigned patients into two broad stages: early stage (Ann-Arbor stages I and II) and late stage (Ann-Arbor stages III and IV). Combining the studies, there were 439 patients, and over 98% of these were late-stage

**Table 1:** Patient Characteristics: Frontline Treatment with R-CVP and R-CHOP

Variable (N; %)	R-CHOP N = 223 Hiddemann et al [5]	R-CVP N = 162 Marcus et al [7]	Total N = 385	P-value
Sex	M:88; 39.5% F: 135; 60.5%	M: 88; 54.3% F: 74; 45.7%	M: 176; 45.7% F: 209; 54.3%	.004
Age>60	82; 36.8%	41; 25.3%	123; 31.9%	.017
ECOG>1	16; 7.2%	4;2.5%	20; 5.2%	.040
B-symptoms	80; 35.9%	65; 40.1%	145; 37.7%	.396
Marrow involvement	136; 61.0%	103; 63.6%	239; 62.1%	.605
IPI> 2	42; 18.8%	21; 13.0%	63; 16.4%	.212
Stage	I/II: 0; 0% III/IV: 223; 100%	I/II: 2; 1.2% III/IV: 160; 98.8%	I/II: 2; 0.5% III/IV: 383; 99.5%	.342
Elevetaed LDH	51; 22.9%	39; 24.1%	90; 23.4%	.783

**Table 2:** Patient Characteristics: Frontline and Re-treatment with R-CHOP and R-CVP

Variables (N; %)	R-CHOP N = 277	R-CVP N = 162	Total N = 439	p-value
Sex	M: 119; 43.0% F: 158; 57.0%	M: 88; 54.3% F: 74; 45.7%	M: 207; 47.2% F: 232; 52.8%	.02
Age > 60	99; 35.7%	41; 25.3%	140; 31.9%	.02
Stages	I/II: 4; 1.4% III/IV: 273; 98.6%	I/II: 2; 1.2% III/IV: 160; 98.8%	I/II: 6; 1.4% III/IV: 433; 98.6%	.86

### Treatment efficacy

The main characteristics of the four studies are shown in table 3, where it is seen that the inclusion criteria were similar across all studies. The R-CHOP and R-CVP arms used identical medication dosing and treatment protocols, with the exception that Adriamycin was not included in the R-CVP regimen whereas it was used in the three R-CHOP regimens. Three of the studies' definitions of CR and PR conformed to the International Working Group Criteria, and the study by Czuczman et al. used similar characteristics.

**Table 3:** Main Characteristics of the Analyzed Trials

**Study**	**Patient Population**	**Treatment** **(N)**	**CR** **(n; %)**	**PR** **(n; %)**	**OR** **(n; %)**	**Criteria**
Czuczman et al [4]: Prospective; Single treatment group; Intent to treat trial	N-40; 18 years and older with CD 20+ follicular lymphoma	R-CHOP N = 40	22; 55%	16; 40%	38; 95%	Table 1
Hiddemann W et al. [5]: Prospective, randomized, non-crossover, open label multicenter phase 3 trial	N = 428; 18 years and older with untreated advanced stage follicular lymphoma	R-CHOP (N = 223) vs. CHOP (N = 205)	44; 20% 35; 17%	170; 77% 150; 73%	214; 96% 185; 90%	IWG[13]
Domingo-Domenech et al. [6]: Prospective, non-randomized, non-crossover, open label multicenter phase 2 trial	N = 16; 18 to 70 years age with CD 20+ follicular lymphoma	R-CHOP (N = 16)	12; 75%	2; 13%	14; 88%	IWG [13]
Marcus R et al. [7]: Prospective, randomized, non-crossover trial	N = 321; 18 years or older, untreated CD 20+ follicular lymphoma	R-CVP (N = 162) vs. CVP (N = 159)	66; 41% 16; 11%	65; 40% 74; 47%	131; 81% 90; 57%	IWG[13]

Data from all four trials were collected and the response rates (CR, PR, and CR+PR) yielded by R-CVP and R-CHOP were summarized and evaluated. The first analysis compared R-CHOP vs. R-CVP for the frontline treatment of follicular lymphoma (Table 4). Patients who underwent R-CVP therapy had a significantly higher chance of attaining CR (41%) than patients treated with R-CHOP (20%) (*P *< 0.001). By contrast, significantly more patients showed PR under R-CHOP (76%) than under R-CVP (40%) (*P *< 0.001), and R-CHOP showed better total CR+PR (96% vs. 81%, *p *<0.001).

**Table 4:** Response of FL to R-CVP and R-CHOP as Frontline Agents

Variable	R-CHOP N = 223	R-CVP N = 160	p-value
CR (n; %)	44; 19.7%	66; 41.3%	< .001 Favors R-CVP
PR (n; %)	170; 76.2%	65; 40.6%	< .001 Favors R-CHOP

Total Response (CR+PR) (n; %)	214; 96.0%	131; 81.9%	< .001 Favors R-CHOP

The second analysis revealed similar findings when comparing R-CVP to R-CHOP (Table 5) excepting that with the additional studies on the R-CHOP side, the CR advantage of R-CVP over R-CHOP (41% vs. 32%) was only marginally significant (*p *= 0.055).

**Table 5:** Response of FL to R-CVP and R-CHOP as Frontline and Re-treatment Agents

Variable	R-CHOP N = 277	R-CVP N = 160	p-value
CR (n; %)	89; 32.1%	66; 41.3%	.055 R-CHOP and R-CVP not significant
PR (n; %)	177; 63.9%	65; 40.6%	< .001 Favors R-CHOP

Total Response (CR+PR) (n; %)	266; 96.0%	131; 81.9%	< .001 Favors R-CHOP

These trends can also be seen in the odds ratios comparing the two treatment modalities. For both studies, R-CVP was superior to R-CHOP when evaluating only for complete response. Odds ratios of 2.86 (95% CI: 1.81–4.51) in the first analysis and 1.48 (95% CI: .991–2.22) in the second analysis favored R-CVP over R-CHOP. However, for overall response (complete response + partial response), R-CHOP was superior, with odds ratios of 5.45 (95% CI: 2.51 – 11.83) and 5.54 (95% CI: 2.69 – 11.40), for the first and second analyses, respectively.

## Discussion

Anthracyclines are always included in the treatment regimen for diffuse large cell lymphoma, but the role of anthracyclines in treating patients with symptomatic FL is not clear. Both R-CHOP and R-CVP are used frequently in the management of symptomatic patients with FL. Hiddemann et al. [13] illustrated current advances in treatment modalities for follicular lymphoma including the use of chemotherapy, radio-therapy, monoclonal antibodies, vaccine strategies, and stem cell transplants. Liu et al. [14] had highlighted increased survival time over the past 25 years for those who have stage 4 FL with advances in treatment options. It is still not clear whether addition of anthracyclines in the therapy for FL confers any benefit.

In our analyses we tried to answer the question whether addition of anthracyclines to the chemotherapeutic regimen improves response in patients with FL. To compare the response rates between R-CHOP and R-CVP in a prospective fashion would require a multi-year multicenter trial involving a large number of patients. For example, using nQuery 5.0, one can determine the sample size per treatment group if the hypothesized treatment difference is that obtained in the current study between R-CHOP and R-CVP for CR+PR (96% vs. 81%). Assuming 80% power using 2-tailed Fisher's Exact test, with significance at *p *< 0.05, the trial would require about 75 patients per treatment group, not counting replacement of patients with incomplete data. With this in mind, we conducted the current meta-analysis of all relevant literature comparing the two treatment options, the primary outcome measures being CR, PR, or CR+PR. Our sources were the commonly used search engines and the abstracts of the American Society of Hematology. In the first analysis, comparing R-CHOP and R-CVP as frontline agents, only two pertinent articles could be retrieved, whereas four were available for the second analysis. This paucity of studies underlines the difficulty inherent in systemically comparing response rates in patients with indolent diseases treated with two different but very effective regimens.

As shown in table 4, patients treated with frontline R-CVP had a much higher chance of developing a CR compared to those with frontline R-CHOP. However, when combined with studies of relapsed or previously treated patients, the difference–still favoring R-CVP–became only marginally significant. One possible explanation for this result is that the patients treated with R-CHOP had higher ECOG scores, were older, and were women. On the contrary, in both the analyses, the probability of achieving an overall response (CR or PR) was significantly higher when patients were treated with R-CHOP as opposed to R-CVP. With the data presented, it would seem that patients would fare better overall with R-CHOP, for 96% experienced some form of response in both analyses, significantly higher than the 81% found for patients treated with R-CVP. However it is unclear and we were unable to obtain usable data to test whether a response of any type necessarily correlates with increased survival.

Multiple observations need to be made about the present data. The most obvious is that the definitions for CR and PR are not uniform across studies. Second, we could retrieve only one prospective study involving R-CVP [7] in the treatment of FL. Third, the high CR rates reported by Czuczman et al. [4] and Domingo-Domenech et al. [6] were not supported by Hiddemann et al. [5]. In the study by Czuczman et al. [4] the number of patients treated with R-CHOP was 38 and in Domingo-Domenech et al. [6] was 16, approximately 20% of the total patient population treated with R-CHOP in our analyses. Hence, the CR rate from this R-CHOP group was clearly lower than R-CVP. From the combined data it would seem evident from both studies that R-CVP was overall inferior to R-CHOP. This difference could be from selection bias and non-comparability of the study subjects.

No data were located for comparing R-CHOP vs. R-CVP for relapsed patients with follicular lymphoma, and no data for previously treated and relapsed patients on R-CVP. Finally, it was unfortunate that for the second analysis only age, sex, and stage were reported as demographic and clinical variables in all four studies. We had to ignore the values for patients' performance status, bone marrow involvement, ECOG status, LDH status, B-symptoms, and IPI because none of these variables was available for all four studies in the analysis. Editors may wish to require future studies to report such fundamental variables not only to satisfy readers' immediate interests but as well with an eye towards future meta-analyses.

For indolent disease like follicular lymphoma, response may not be an adequate end-point. Although some recent studies suggest the importance of CR for survival, the progression-free survival or time to treatment failure (TTF) could be more relevant and could avoid the bias of measurement. Marcus et al [7] investigated the addition of rituximab to CVP (R-CVP) therapy compared with CVP therapy alone and showed a significant advantage for R-CVP for remission rate (81% vs. 57%; *P *< .001), TTF (27 months vs. 7 months; *P *< .001), and time to next therapy (median not reached vs. 12 months; *P *< .001). However, remission rates and TTF achieved by R-CVP appear comparable to the results obtained by CHOP alone. A substantially better outcome seems to be achieved by R-CHOP. Adverse effects, particularly severe granulocytopenia, were less frequently encountered after CVP (14%) or R-CVP (24%) than after CHOP (53%) or R-CHOP (63%).

An unavoidable weakness of any meta-analysis is its inability to perform multivariate analyses that might throw light on the importance of various potentially confounding variables for the overall outcome, in ways not avail to any of the constituent studies because of their limited sample sizes.

QUOROM provides a system for rating studies to be included in meta-analyses of randomized clinical trials. We did not use this system, for we wished to incorporate all available data. We judged all four cited articles as equally relevant in providing accurate data for our purposes. Other studies utilizing G-CSF with R-CHOP for the treatment of FL, such as by Niitsu et al.[15], were excluded because they attempted to use G-CSF as a treatment modality, and responses changed depending on the dose of G-CSF provided. As with any paper, much information was omitted from our study and one should not use this article as a crutch when determining the appropriate chemotherapeutic protocol for a patient. Profiles of side effects of Adriamycin were not evaluated, nor could we provide a correlation between specific responses and length of survival or cost of treatment. One should always evaluate specific cases when deciding the treatment protocol appropriate for the individual.

The international PRIMA study testing the efficacy of maintenance therapy by rituximab may provide important data in the field of the best induction in patients with follicular lymphoma.

In summary, we conclude that treatment for patients with FL should be individualized. R-CHOP and R-CVP protocols can both achieve excellent overall response. In patients with known cardiac history, omission of anthracyclines is reasonable, and R-CVP provides a very good CR rate. In younger patients with FL where cumulative cardio-toxicity may be of importance in the long term and in whom future stem cell transplantation is an option, R-CVP may be a more appealing option. How the response rates translate to survival is not known and certainly needs to be further clarified in prospectively designed long-term follow-up studies.

## Competing interests

The authors declare that they have no competing interests.

## Authors' contributions

Both authors helped in design, data collection, manuscript writing, and review of this article.
